# Molecular Characterization of Extended-Spectrum Beta-Lactamase-Producing Enterobacterales Among Cancer Patients at the Mbarara Regional Referral Hospital, Southwestern Uganda

**DOI:** 10.7759/cureus.114711

**Published:** 2026-08-18

**Authors:** Henry Zamarano, Jacob Stanley Iramiot, Edgar Mulogo, Natumanya Debrah, Catherine Khakasa, Benson Musinguzi, Vicent Mwesigye, Iving Mumbere, Ivan Muhwezi, Kawuma Simon, Orikiriza Patrick

**Affiliations:** 1 Department of Microbiology, Uganda Protestant Medical Bureau, Kampala, UGA; 2 Department of Microbiology and Immunology, Busitema University, Mbale, UGA; 3 Department of Community Health, Mbarara University of Science and Technology, Mbarara, UGA; 4 Faculty of Computing and Informatics, Mbarara University of Science and Technology, Mbarara, UGA; 5 Microbiology, Mbarara University of Science and Technology, Mbarara, UGA; 6 Department of Microbiology, Muni University, Arua, UGA; 7 Medical Laboratory Science/Parasitology/Neglected Tropical Diseases, Mbarara University of Science and Technology, Mbarara, UGA; 8 Pharmacy, Makerere University, Kampala, UGA; 9 Microbiology, Makerere University, Kampala, UGA; 10 Software and Informatics Engineering, Mbarara University of Science and Technology, Mbarara, UGA; 11 Microbiology, University of Global Health Equity, Kigali, RWA

**Keywords:** and uganda, cancer patients, ctx-m, esbl-producing enterobacterales, molecular characterization, resistance genes

## Abstract

Background and objective

Extended-spectrum β-lactamase-producing *Enterobacterales* (ESBL-PE) are an important cause of antimicrobial-resistant infections among immunocompromised patients. Cancer patients are vulnerable because of frequent hospital exposure, invasive procedures, chemotherapy-associated immunosuppression, and antibiotic use. This study aimed to determine the molecular characteristics of ESBL-PE among cancer patients at Mbarara Regional Referral Hospital (MRRH), Southwestern Uganda.

Methods

A laboratory-based cross-sectional study was conducted at the Microbiology and Molecular Laboratory of Mbarara University of Science and Technology (MUST). The study used archived clinical *Enterobacterales* isolates recovered from clinical specimens collected from inpatient and outpatient cancer patients receiving care at MRRH between June and November 2025. The isolates had been identified using standard microbiological methods, and ESBL-PE were confirmed using the double-disk synergy test (DDST). Molecular detection of ESBL and other antimicrobial resistance-associated genes was performed using conventional polymerase chain reaction (PCR) with gene-specific primers. Target genes included *blaCTX-M*, *blaTEM*, *blaSHV*, *blaOXA-1*, *AmpC-blaCITM*, *blaKPC*, *blaNDM*, *blaVIM*, *blaIMP*, *blaOXA-48*, *AAC(6’)-Ib*, *mcr-1*, *qnr*, *FosA*, and *blaPER*. PCR products were separated by agarose gel electrophoresis and visualized under ultraviolet illumination. Data were entered into a database and analyzed using frequencies and percentages to summarize the distribution of resistance genes among isolates.

Results

A total of 281 isolates were analyzed. *Escherichia coli* (*E. coli*) was predominant (155/281, 55.2%), followed by *Klebsiella pneumoniae* (*K. pneumoniae*) (85/281, 30.2%). *CTX-M* was the most frequently detected resistance gene (106/281, 37.7%), followed by *TEM* (81/281, 28.8%), *SHV* (26/281, 9.3%), and *AAC* (24/281, 8.5%). At least one target resistance gene was detected in 170/281 (60.5%) isolates, while 111/281 (39.5%) had no target genes detected within the PCR panel. Multi-gene carriage occurred in 76/281 (27.0%), with *TEM* + *CTX-M* as the most common co-carriage pattern (25/281, 8.9%). Carbapenemase-associated genes remained uncommon.

Conclusions

The findings of this study describe the distribution of ESBL-PE in this population and highlight the importance of continued surveillance of resistant *Enterobacterales* among patients receiving cancer care.

## Introduction

Extended-spectrum β-lactamase-producing *Enterobacterales* (ESBL-PE) are a major contributor to the global antimicrobial resistance (AMR) crisis because they compromise the effectiveness of third-generation cephalosporins and are frequently multidrug-resistant [1]. Globally, bacterial AMR was estimated to be directly responsible for 1.27 million deaths and associated with 4.95 million deaths in 2019, with *Escherichia coli (E. coli)* and *Klebsiella pneumoniae (K. pneumoniae)* among the leading bacterial contributors to this burden [2,3]. The global intestinal carriage of ESBL-producing *E. coli* has also increased substantially, with pooled carriage estimates of 17.6% among healthy individuals and 21.1% among inpatients in healthcare settings [4]. In Africa, pooled estimates suggest a high ESBL-PE burden, with one meta-analysis reporting an overall pooled prevalence of 38% across the continent [5]. In East African hospitals, Sonda et al. reported a pooled proportion of ESBL-PE of 42%, with country-level estimates as high as 62% in Uganda, 47% in Kenya, and 30% in Ethiopia [6]. Uganda’s national AMR surveillance report further showed high levels of third-generation cephalosporin-resistant *Enterobacterales*, ranging from 49% to 55%, underscoring the local public health relevance of ESBL-associated resistance [7].

Cancer patients represent a particularly vulnerable population for ESBL-PE infection because malignancy and its treatment often impair host immunity. Chemotherapy-induced neutropenia, mucosal barrier injury, gastrointestinal colonization, prolonged hospitalization, broad-spectrum antibiotic exposure, invasive devices, and repeated healthcare contact all contribute to an increased risk of infection with resistant Gram-negative organisms [8,9]. A systematic review and meta-analysis among patients with malignancy found that the pooled prevalence of gastrointestinal ESBL-PE colonization was 19%, and that colonized cancer patients were nearly 13 times more likely to develop ESBL-PE bloodstream infection during hospitalization than non-colonized patients [8]. In Uganda, Lubwama et al. reported that multidrug-resistant Gram-negative bacteria were a major cause of bacteremia among febrile cancer patients, and later demonstrated that *CTX-M* was the dominant ESBL-encoding gene among *Enterobacterales* from hematological cancer patients with febrile neutropenia and bacteremia [10,11].

These findings justify targeted molecular surveillance of ESBL-PE among cancer patients, who are both clinically vulnerable and likely to experience poor outcomes when empirical therapy fails. Uganda’s cancer burden is on the rise, with national estimates indicating 34,008 new cancer cases and 22,992 cancer-related deaths in 2020 [12,13], while available population-based cancer registry data cover only a small fraction of the national population. Conducting this study at Mbarara Regional Referral Hospital (MRRH) therefore provides evidence from a high-volume regional cancer-care setting outside the Uganda Cancer Institute, contributing data from a geographically important but understudied population. Despite the high ESBL-PE burden in Uganda and the recognized vulnerability of cancer patients, important gaps remain in the local molecular epidemiology. Most Ugandan cancer-patient ESBL data have come from the Uganda Cancer Institute and have focused mainly on bloodstream infections, hematological malignancies, or specific ESBL genes.

There is limited evidence from regional referral cancer-care settings such as MRRH, especially regarding the distribution of ESBL and other resistance-associated genes across diverse *Enterobacterales* isolates from cancer patients. This gap is important since molecular characterization identifies the dominant resistance genes, detects co-carriage patterns, informs empirical treatment decisions, supports antimicrobial stewardship, and strengthens infection prevention and control planning. Generating local molecular data is therefore essential for guiding context-specific cancer-care antibiotic policies and facilitating the early detection of emerging resistance mechanisms, including carbapenemase-associated genes. This study aimed to determine the molecular characterization of ESBL-PE, including the co-carriage of ESBL and carbapenemase-associated resistance genes, among cancer patients at MRRH in southwestern Uganda.

## Materials and methods

Study design

This was a laboratory-based cross-sectional study conducted at the Microbiology and Molecular Biology Laboratory of Mbarara University of Science and Technology (MUST).

Study site and setting

The study was conducted in the outpatient department and inpatient cancer wards of MRRH, which registers approximately 3,000 new cancer cases annually, about nine new cases per day as of March 2018 [14]

Study population and sample collection

ESBL-PE isolates were identified from clinical specimens, including blood, urine, stool, sputum, cerebrospinal fluid (CSF), swabs, and aspirates, collected from cancer patients receiving care as inpatients or outpatients at MRRH who presented with signs and symptoms suggestive of bacterial infections between June and November 2025. Molecular characterization of AMR genes was performed on phenotypically confirmed ESBL-PE isolates. Phenotypic confirmation of ESBL production was carried out using the double-disk synergy test (DDST). To avoid duplication, only the first phenotypically confirmed ESBL-PE isolate recovered from each participant during the study period was included in the analysis.

Ethical considerations

Written informed consent was obtained from all adult participants before enrolment. For participants younger than 18 years, written informed consent was obtained from a parent or legal guardian, with assent obtained from minors where appropriate. Ethical approval was granted by the MUST Faculty of Medicine Research Review Committee and the Institutional Research Ethics Committee (Approval No. MUST-2024-1684), and administrative clearance was obtained from the Director of MRRH.

Samples size

The minimum sample size was calculated using the Kish formula, assuming a 21% prevalence of ESBL-PE based on a previous study [15], a 95% confidence level (CI), and a 5% margin of error [16]. The calculated sample size was 255, and 10% was added to account for potential losses, giving a final minimum sample size of 281 ESBL-PE isolates.

Phenotypic ESBL detection and selection for molecular analysis

*Enterobacterales* isolates were identified based on colony morphology, Gram staining characteristics, and conventional biochemical tests, including indole production, urease activity, citrate utilization, motility, carbohydrate fermentation, and lysine decarboxylation. Species identification was confirmed using the Analytical Profile Index (API). All confirmed *Enterobacterales* isolates were screened for ESBL production using the Kirby-Bauer disk diffusion method in accordance with the Clinical and Laboratory Standards Institute (CLSI) 2025 guidelines [17]. Isolates demonstrating reduced susceptibility to third-generation cephalosporins were subjected to phenotypic confirmation using the DDST.

For the DDST, a standardized bacterial suspension equivalent to a 0.5 McFarland standard was inoculated onto Mueller-Hinton agar using a sterile swab to produce a uniform bacterial lawn. A ceftazidime (30 μg) disk and a ceftazidime-clavulanic acid (30/10 μg) disk were placed 25 mm apart (center-to-center), and the plates were incubated aerobically at 35 ± 2 °C for 16-18 hours. An increase of ≥ 5 mm in the inhibition zone diameter around the ceftazidime-clavulanic acid disk compared with the ceftazidime disk alone was interpreted as phenotypic confirmation of ESBL production, in accordance with CLSI 2025 guidelines. Only isolates that were phenotypically confirmed as ESBL-PE were included in the molecular analysis. DNA extraction and polymerase chain reaction (PCR) assays were subsequently performed to detect selected ESBL-associated resistance genes.

Quality control

Standard operating procedures were followed throughout isolate recovery, bacterial identification, phenotypic ESBL confirmation, antimicrobial susceptibility testing, and molecular analysis. Culture media were quality-checked for sterility and performance before use. Reference strains, including* Escherichia coli *ATCC 25922 and *Klebsiella pneumoniae *ATCC 700603, were used for quality control of phenotypic testing procedures. Positive and negative controls were included in each PCR run to ensure accuracy and reliability of molecular results.

Identification of ESBL and other resistance genes

Conventional PCR was used to detect ESBL resistance-associated genes using gene-specific forward and reverse primers. The target genes included *blaTEM, blaSHV, blaCTX-M, blaOXA-1, AmpC-blaCITM, blaKPC, blaNDM, blaVIM, blaIMP, blaOXA-48, AAC(6’)-Ib, mcr-1, qnr, FosA and blaPER. *All primers were adopted from previously published, validated assays targeting the specific resistance-gene sequences of interest, and primer specificity and expected amplicon size were reconfirmed for this study using known gene-positive and gene-negative control strains included alongside the study isolates in every PCR run.

DNA extraction

DNA was extracted from bacterial colonies using a heat-lysis method where bacterial colonies were washed twice with 1× Tris-EDTA (TE) buffer and pelleted by centrifugation. The pellet was then resuspended in 100 µL of TE elution buffer and vortexed for one minute to ensure thorough homogenization. The suspension was incubated at 95 °C for 15 minutes in a dry heating block to facilitate cell lysis and release of genomic DNA. Following incubation, the lysate was centrifuged at 15,000 rpm for five minutes to pellet cellular debris. The resulting supernatant containing the extracted DNA was carefully transferred to a sterile microcentrifuge tube, while the pellet and original tube were discarded. The extracted DNA was subsequently stored at −20° C.

Primer sequences used for PCR amplification of antimicrobial resistance genes

Each of the open reading frames of specific targets was amplified using forward and reverse primers, each with a unique sequence, concentration, and annealing temperature, as shown in Table 1.

**Table 1:** Primer sequences used for PCR amplification of antimicrobial resistance genes PCR: polymerase chain reaction

Gene	Primer	Primer Sequences (5’-3’)	Amplicon size (bp)	Annealing temperature (°C)	Primer concentration (µM)
FosA	Forward	GAGCGTGGCGTTTTATCAGC	267	55	10
	Reverse	GCCTCGCACTACTTCCTCGA			
blaTEM	Forward	CATTTCCGTGTCGCCCTTATTC	800	55	5
	Reverse	CGTTCATCCATAGTTGCCTGAC			
blaSHV	Forward	ATG CGT TAT ATT CGC CTG TG	747	55	5
	Reverse	TGC TTT GTT ATT CGG GCC AA			
blaCTX-M	Forward	CGCTTTGCGATGTGCAG	550	55	25
	Reverse	ACCGCGATATCGTTGGT			
*blaOXA-*1	Forward	TTTTCTGTTGTTTGGGTTTT	427	55	10
	Reverse	TTTCTTGGCTTTTATGCTTG			
AmpC-blaCITM	Forward	TGG CCA GAA CTG ACA GGC AAA	465	55	10
	Reverse	TTT CTC CTG AAC GTG GCT GGC			
blaKPC	Forward	TCGAACAGGACTTTGGCG	201	55	10
	Reverse	GGAACCAGCGCATTTTTGC			
blaNDM	Forward	GCATAAGTCGCAATCCCCG	237	55	10
	Reverse	CTTCCTATCTCGACATGCCG			
blaVIM	Forward	GTTTGGTCGCATATCGCAAC	382	55	10
	Reverse	AATGCGCAGCACCAGGATAG			
blaIMP	Forward	GAAGGCGTTTATGTTCATAC	587	55	10
	Reverse	GTAAGTTTCAAGAGTGATGC			
*blaOXA-*48	Forward	GCGTGGTTAAGGATGAACAC	585	55	50
	Reverse	CATCAAGTTCCAACCCAACCG			
AAC(6′)-Ib	Forward	TTGCGATGCTCTATGAGTGGCTA	482	55	10
	Reverse	CTCGAATGCCTGGCGTGTTT			
*mcr-*1	Forward	CGGTCAGTCCGTTTGTTC	309	55	10
	Reverse	CTTGGTCGGTCTTGTAGCC			
qnr	Forward	ATTTCTCACGCCAGGATTTG	516	55	25
	Reverse	GATCGGCAAAGGTTAGGTCA			
blaPER	Forward	AATTTGGGCTTAGGGCAGAA	924	55	25
	Reverse	ATGAATGTCATTATAAAAGC			

PCR reaction preparation

PCR assays were performed in a final reaction volume of 25 µL. Each reaction mixture contained 2.5 µL of 10× PCR buffer, 0.5 µL of deoxynucleotide triphosphates (dNTPs), 0.5 µL of Taq DNA polymerase (New England Biolabs, Ipswich, MA), 0.5 µL of forward primer, 0.5 µL of reverse primer, 3.0 µL of template DNA, and 17.5 µL of RNase-free water. PCR master mixes were prepared on ice to minimize nonspecific amplification and contamination. Where appropriate, duplex PCR assays were performed by incorporating two primer pairs into a single reaction mixture

PCR amplification

PCR amplification was carried out using a Multigene Optimax Thermal Cycler. The cycling conditions consisted of an initial denaturation step at 95 °C for three minutes, followed by 40 cycles of denaturation at 95 °C for 30 seconds, primer annealing at 55 °C for 30 seconds, and extension at 72 °C for one minute. A final extension step was performed at 72 °C for five minutes to ensure complete amplification of all PCR products. The amplified products were then held at 4 °C until further analysis.

Agarose gel electrophoresis

**Figure 1:**
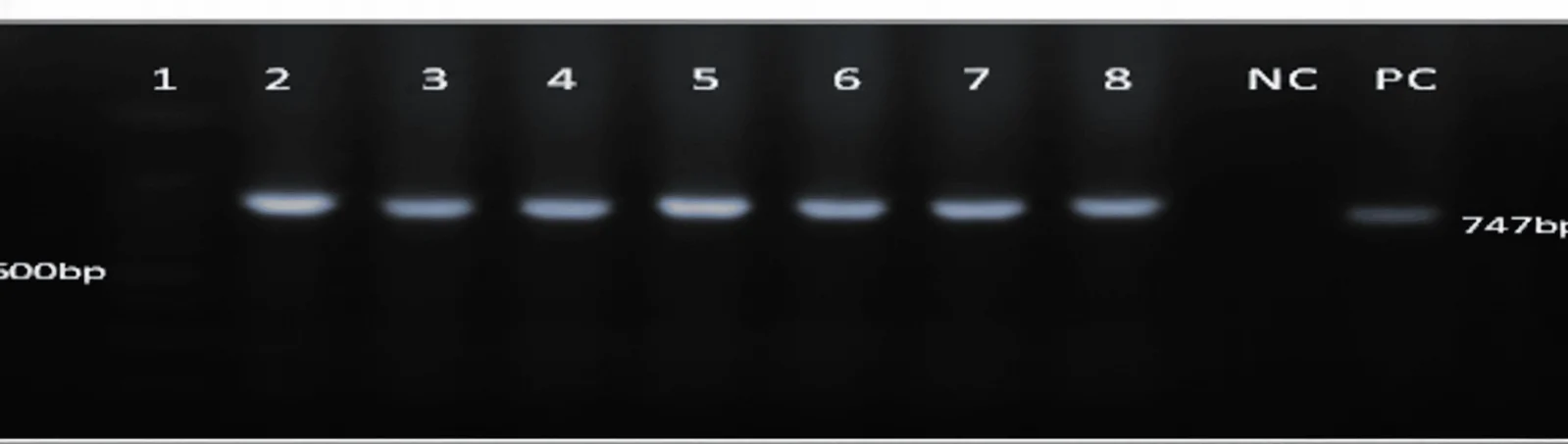
Gel image showing the successful amplification of the SHV target, yielding an expected product size of approximately 747 bp Lane 1: DNA 100bp ladder. Lanes 2-8: Amplified PCR products from experimental samples. Lane NC: Negative Control (no-template control showing no amplification). Lane PC: Positive Control (showing the expected 747 bp band)

**Figure 2:**
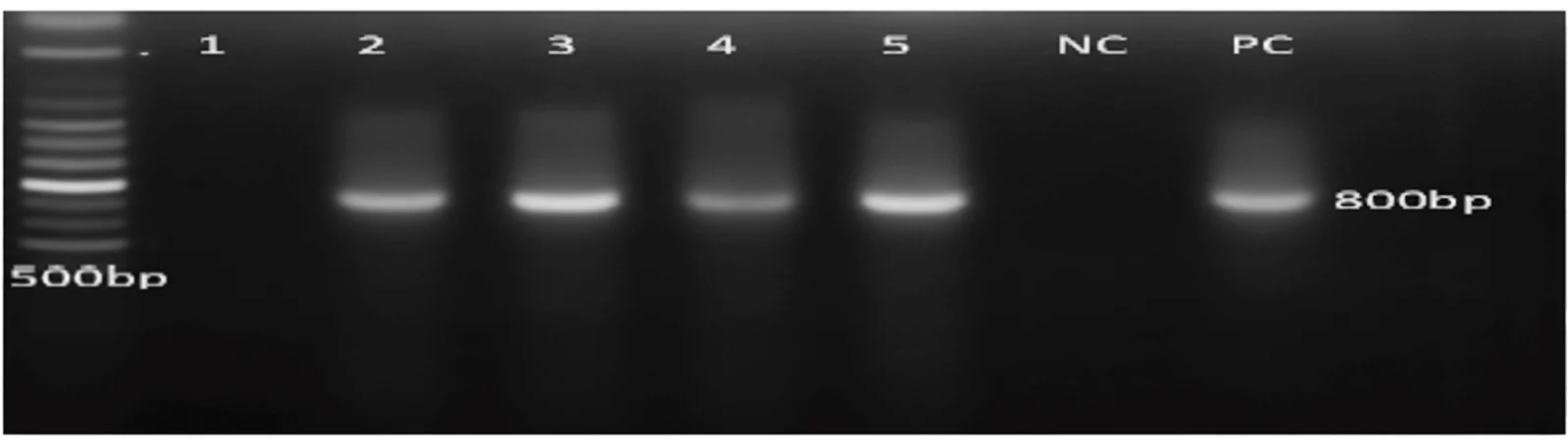
Gel image showing the successful amplification of the TEM target, yielding an expected product size of approximately 800 bp Lane 1: DNA 100bp ladder. Lanes 2-5: Amplified PCR products from experimental samples. Lane NC: Negative Control (no-template control showing no amplification). Lane PC: Positive Control (showing the expected 800 bp band)

**Figure 3:**
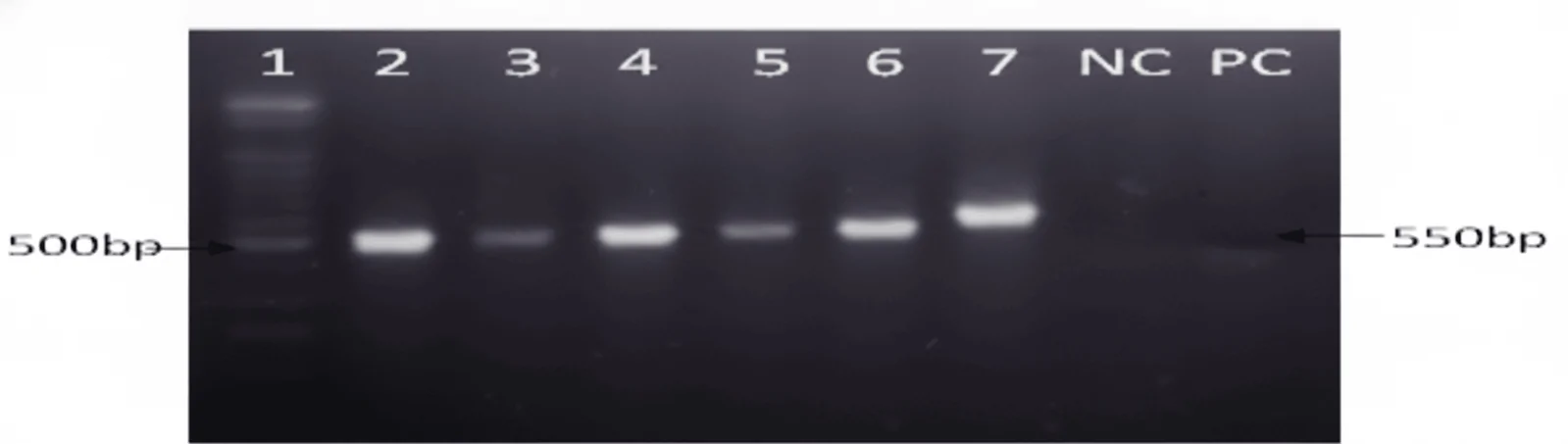
Gel image showing the successful amplification of the CTX target, yielding an expected product size of approximately 550 bp Lane 1: DNA 100bp ladder. Lanes 2-7: Amplified PCR products from experimental samples. Lane NC: Negative Control (no-template control showing no amplification). Lane PC: Positive Control (showing the expected 550 bp band)

PCR amplicons were analyzed by agarose gel electrophoresis, where a 1.5% agarose gel was prepared in 1× Tris-Borate-EDTA (TBE) buffer and supplemented with 5 µL of DSView™ nucleic acid stain (Cat. No. M7011). Amplified DNA products were mixed with 6× loading dye (GDSBio, Lot 050) and loaded onto the gel alongside a 100-bp DNA ladder (GDSBio, Lot 076) for size estimation. Electrophoresis was performed in 1× TBE buffer at 200 V and 80 mA for one hour. Following electrophoresis, DNA bands were visualized and documented using a Gene-Flash transilluminator as shown in Figures 1-3 below. A band was scored as positive only where a clear, distinct band matched both the expected amplicon size for that gene (Table 1) and the corresponding positive control band; faint, smeared, or off-size bands were classified as indeterminate, and the PCR reaction was repeated before a final result was assigned. Bands that remained faint or ambiguous after repeat testing were recorded as gene-negative for that isolate

Statistical analysis

The analytic unit was the bacterial isolate. Data were cleaned and organized in Microsoft Excel. Bacterial isolate names were harmonized before analysis to combine spelling and formatting variants. Categorical variables were summarized using frequencies and percentages. The distribution of bacterial isolates was reported as n (%) out of the total number of isolates. For gene-distribution analysis, gene positivity was summarized by bacterial isolate, with percentages calculated using the total number of isolates within each bacterial species as the denominator. Overall gene prevalence was calculated using the total isolate count as the denominator. Molecular co-carriage patterns were generated by identifying all resistance genes detected in each isolate and grouping isolates according to single-gene, multi-gene, or no-target-gene profiles. A heat map was used to visualize the percentage distribution of resistance genes across bacterial isolates, while a bar chart was used to present the overall prevalence of each detected resistance gene.

## Results

Demographic and clinical characteristics of participants from whom bacterial samples were identified

A total of 281 ESBL-PE isolates were recovered from 281 cancer patients. The largest proportion of these cancer patients belonged to the 25-44-year age group (91/281; 32.4%), followed by those aged 45-59 years (79/281; 28.1%). Children aged 0-14 years accounted for 43/281 (15.3%), while participants aged 60-74 years and ≥75 years represented 39/281 (13.9%) and 25/281 (8.9%), respectively. The 15-24 years age group was the least represented, comprising only 4/281 (1.4%) of participants. Overall, nearly two-thirds (60.5%) of ESBL-PE isolates were recovered from participants aged 25-59 years, indicating that the burden of bacterial isolation was highest among middle-aged adults.

Females constituted the majority of participants, accounting for 175/281 (62.3%), whereas males represented 106/281 (37.7%), resulting in a female-to-male ratio of approximately 1.7:1. Regarding cancer diagnoses, carcinomas were the predominant malignancy, accounting for 168/281 (59.8%) of participants. This was followed by lymphomas (35/281; 12.5%) and plasma cell dyscrasias (23/281; 8.2%). Less common malignancies included sarcomas (16/281; 5.7%), embryonal tumors (14/281; 5.0%), leukemias (14/281; 5.0%), blastomas (8/281; 2.8%), and gliomas (3/281; 1.1%). These findings indicate that most bacterial isolates were recovered from patients with solid tumors, particularly those with carcinomas.

Urine was the most common specimen source, contributing 57/281 (20.3%) of all ESBL-PE isolates. Stool specimens accounted for 46/281 (16.4%), while swabs contributed 43/281 (15.3%). Blood and sputum each yielded 41/281 (14.6%) isolates, followed by aspirates (33/281; 11.7%) and CSF (20/281; 7.1%). Overall, urinary and gastrointestinal specimens (urine and stool) together accounted for more than one-third (36.7%) of all specimens yielding ESBL-PE isolates, as shown in Table 2. Most participants were hospitalized inpatients (191/281; 68.0%), whereas 90/281 (32.0%) were managed as outpatients. This distribution suggests that ESBL-PE isolates were more frequently recovered from hospitalized cancer patients, who are generally at greater risk of healthcare-associated bacterial infections due to prolonged hospitalization, invasive procedures, immunosuppression, and frequent antimicrobial exposure. 

**Table 2:** Demographic and clinical characteristics of participants from whom bacterial samples were identified (N = 281)

Variable	Level	Frequency (n)	Proportion (%)
Age group (years)	0-14	43	15.3
	15-24	4	1.4
	25-44	91	32.4
	45-59	79	28.1
	60-74	39	13.9
	≥ 75	25	8.9
Gender	Female	175	62.3
	Male	106	37.7
Cancer type	Carcinoma	168	59.8
	Lymphoma	35	12.5
	Sarcoma	16	5.7
	Blastoma	8	2.8
	Embryonal tumor	14	5.0
	Glioma	3	1.1
	Leukemia	14	5.0
	Plasma cell dysplasia	23	8.2
Specimen type	Urine	57	20.3
	Stool	46	16.4
	Swabs	43	15.3
	Blood	41	14.6
	Sputum	41	14.6
	Aspirates	33	11.7
	Cerebrospinal fluid	20	7.1
Admission status	Inpatients	191	68.0
	Outpatient	90	32.0

Distribution of ESBL-producing *Enterobacterales*

A total of 281 phenotypically confirmed ESBL-PE isolates recovered from cancer patients at MRRH underwent molecular characterization. *E. coli *was the predominant ESBL-PE, accounting for 155/281 (55.2%) of all isolates. This was followed by *K. pneumoniae*, which comprised 85/281 (30.2%) of isolates. Together, these two clinically important pathogens represented 240/281 (85.4%) of all ESBL-PE isolates, indicating that they were the principal reservoirs of ESBL production among cancer patients in this setting. The remaining isolates were recovered at substantially lower frequencies. *Klebsiella oxytoca (K. oxytoca)* and *Enterobacter cloacae (E. cloacae)* each accounted for 15/281 (5.3%) of isolates. *Proteus mirabilis (P. mirabilis)* represented 5/281 (1.8%), while *Klebsiella aerogenes (K. aerogenes),* *Proteus vulgaris (P. vulgaris),* and *Citrobacter freundii (C. freundii) *were less frequently recovered, accounting for 3/281 (1.1%), 2/281 (0.7%), and 1/281 (0.4%), respectively.

Overall, species belonging to the genus *Klebsiella* (*K. pneumoniae, K. oxytoca*, and *K. aerogenes*) collectively accounted for 103/281 (36.7%) of all ESBL-PE isolates, highlighting the important contribution of *Klebsiella* species to the ESBL burden. In contrast, *Proteus* species (*P. mirabilis* and *P. vulgaris*) collectively represented only 7/281 (2.5%), while *E. cloacae* and *C. freundii* together contributed 16/281 (5.7%) of isolates, as shown in Table 3.

**Table 3:** Distribution of ESBL-PE ESBL-PE: extended-spectrum β-lactamase-producing *Enterobacterales*

Bacterial isolate	Frequency (n)	Proportion (%)
Escherichia coli	155	55.2
Klebsiella pneumoniae	85	30.2
Klebsiella oxytoca	15	5.3
Enterobacter cloacae	15	5.3
Proteus mirabilis	5	1.8
Klebsiella aerogenes	3	1.1
Proteus vulgaris	2	0.7
Citrobacter freundii	1	0.4
Total	281	100.0

Distribution of antimicrobial resistance genes among ESBL-producing *Enterobacterales*

A total of 281 ESBL-PE isolates were screened for 12 antimicrobial resistance genes by PCR. Overall,* blaCTX-M* was the most frequently detected resistance gene, occurring in 106/281 (37.7%) isolates. This was followed by *blaTEM* in 81/281 (28.8%),* blaSHV* in 26/281 (9.3%), and the aminoglycoside resistance gene *AAC* in 24/281 (8.5%). Other resistance determinants were detected at lower frequencies, including blaOXA-1 (12/281; 4.3%), *AmpC*-*blaCITM* (7/281; 2.5%),* FosA *(6/281; 2.1%), *KPC* (4/281; 1.4%), *IMP* (2/281; 0.7%), *NDM* (1/281; 0.4%),* VIM* (1/281; 0.4%), and *qnr *(1/281; 0.4%). Neither *PER, OXA*-48, nor *mcr*-1 was detected in any isolate.

Species-specific analysis showed that *E. coli*, the predominant ESBL-producing organism, also harbored the largest absolute number of resistance genes (Table 4). Among the 155 *E. coli i*solates, *blaCTX-M* was detected in 49 (31.6%), followed by *blaTEM* in 42 (27.1%), *AAC* in eight (5.2%), *blaOXA*-1 in six (3.9%), and* blaSHV* in five (3.2%). Sporadic detection of *KPC, NDM, IMP, VIM, FosA, *and *AmpC-blaCITM* was also observed. Among 85* K. pneumoniae* isolates, blaCTX-M was again the predominant gene, detected in 37 (43.5%), followed by blaTEM (27; 31.8%), blaSHV (17; 20.0%), and AAC (15; 17.6%). This species also demonstrated the highest prevalence of *blaSHV*, consistent with the intrinsic association of *SHV *enzymes with *Klebsiella* species.

**Table 4:** Distribution of ESBL genes among Enterobacterales ESBL: extended-spectrum β-lactamase

Bacterial isolate	Total isolates, n	TEM, n (%)	NDM, n (%)	CTX-M, n (%)	SHV, n (%)	FosA, n (%)	AmpC-blaCITM, n (%)	qnr, n (%)	KPC, n (%)	OXA-1, n (%)	IMP, n (%)	AAC, n (%)	VIM, n (%)
Escherichia coli	155	42 (27.1)	1 (0.6)	49 (31.6)	5 (3.2)	4 (2.6)	1 (0.6)	0 (0.0)	3 (1.9)	6 (3.9)	1 (0.6)	8 (5.2)	1 (0.6)
Klebsiella pneumoniae	85	27 (31.8)	0 (0.0)	37 (43.5)	17 (20.0)	1 (1.2)	5 (5.9)	0 (0.0)	0 (0.0)	5 (5.9)	1 (1.2)	15 (17.6)	0 (0.0)
Klebsiella oxytoca	15	3 (20.0)	0 (0.0)	6 (40.0)	1 (6.7)	0 (0.0)	1 (6.7)	0 (0.0)	1 (6.7)	1 (6.7)	0 (0.0)	0 (0.0)	0 (0.0)
Enterobacter cloacae	15	6 (40.0)	0 (0.0)	9 (60.0)	2 (13.3)	1 (6.7)	0 (0.0)	0 (0.0)	0 (0.0)	0 (0.0)	0 (0.0)	1 (6.7)	0 (0.0)
Proteus mirabilis	5	1 (20.0)	0 (0.0)	2 (40.0)	1 (20.0)	0 (0.0)	0 (0.0)	1 (20.0)	0 (0.0)	0 (0.0)	0 (0.0)	0 (0.0)	0 (0.0)
Klebsiella aerogenes	3	2 (66.7)	0 (0.0)	2 (66.7)	0 (0.0)	0 (0.0)	0 (0.0)	0 (0.0)	0 (0.0)	0 (0.0)	0 (0.0)	0 (0.0)	0 (0.0)
Proteus vulgaris	2	0 (0.0)	0 (0.0)	1 (50.0)	0 (0.0)	0 (0.0)	0 (0.0)	0 (0.0)	0 (0.0)	0 (0.0)	0 (0.0)	0 (0.0)	0 (0.0)
Citrobacter freundii	1	0 (0.0)	0 (0.0)	0 (0.0)	0 (0.0)	0 (0.0)	0 (0.0)	0 (0.0)	0 (0.0)	0 (0.0)	0 (0.0)	0 (0.0)	0 (0.0)
Overall	281	81 (28.8)	1 (0.4)	106 (37.7)	26 (9.3)	6 (2.1)	7 (2.5)	1 (0.4)	4 (1.4)	12 (4.3)	2 (0.7)	24 (8.5)	1 (0.4)

Among the less common species, *blaCTX-M *remained the dominant ESBL determinant. It was identified in 9/15 (60.0%) *E. cloacae* isolates, 6/15 (40.0%)* K. oxytoca* isolates, 2/3 (66.7%) *K. aerogenes* isolates, 2/5 (40.0%) *P. mirabilis* isolates, and 1/2 (50.0%) *P. vulgaris* isolates. No target resistance gene was detected in the single *C. freundii* isolate. Although all 281 isolates were phenotypically confirmed as ESBL producers by the DDST, only 170 (60.5%) carried at least one of the targeted resistance genes included in the PCR panel. Consequently, 111/281 (39.5%) isolates showed no detectable target gene (Table 4), demonstrating a substantial phenotypic-genotypic discordance. This discrepancy may reflect the presence of ESBL genes not included in the PCR panel, sequence variations affecting primer binding, low gene copy numbers, or alternative β-lactam resistance mechanisms such as other ESBL families (e.g., Guiana extended-spectrum β-lactamase, Belgium extended-spectrum β-lactamase, Tlahuicas β-lactamase, Brazilian extended-spectrum β-lactamase, *Serratia fonticola* β-lactamase, Vietnamese extended-spectrum β-lactamase), plasmid-mediated or chromosomal *AmpC* β-lactamases, porin loss, or efflux pump overexpression.

Molecular resistance gene patterns of ESBL-PE

The molecular characterization of the 281 ESBL-PE isolates demonstrated substantial diversity in resistance gene carriage patterns. Overall, 170/281 (60.5%) isolates carried at least one of the targeted antimicrobial resistance genes included in the PCR panel, whereas 111/281 (39.5%) had no detectable target gene despite exhibiting a phenotypic ESBL-positive profile. Among the isolates with detectable genes, single-gene carriage was the most common molecular profile, occurring in 94/281 (33.5%) isolates, representing 55.3% (94/170) of all gene-positive isolates. The predominant single-gene pattern was *blaCTX-M* alone, detected in 48/281 (17.1%) isolates, followed by *blaTEM *alone in 28/281 (10.0%) isolates. Single carriage of *AAC, blaSHV, AmpC-blaCITM, blaOXA*-1, *FosA*, and* KPC* was uncommon, each occurring in fewer than 3% of isolates.

In contrast, multi-gene carriage was identified in 76/281 (27.0%) isolates, accounting for 44.7% (76/170) of gene-positive isolates. Dual-gene combinations predominated among multidrug resistance profiles. The most frequent combination was *blaTEM* +* blaCTX-M,* detected in 25/281 (8.9%) isolates, followed by* blaCTX-M *+ *blaSHV *(9/281; 3.2%) and *blaCTX-M* + *AAC* (6/281; 2.1%). Triple-gene combinations were less common, with *blaTEM *+* blaCTX-M *+ *blaSHV* identified in 6/281 (2.1%) isolates. Numerous additional combinations involving *AmpC-blaCITM, blaOXA-*1, *AAC, FosA, KPC, IMP, NDM, VIM,* and *qnr* were detected sporadically, each occurring in only one or two isolates, as seen in Table 5.

**Table 5:** Molecular resistance gene patterns of ESBL-PE (N = 281) ESBL-PE: extended-spectrum β-lactamase-producing *Enterobacterales*

Molecular resistance gene pattern	Frequency (n)	Proportion (%)
No target gene detected	111	39.5
CTX-M	48	17.1
TEM	28	10.0
*TEM *+ *CTX-M*	25	8.9
*CTX-M *+* SHV*	9	3.2
AAC	7	2.5
*CTX-M *+ *AAC*	6	2.1
*TEM *+* CTX-M *+* SHV*	6	2.1
SHV	5	1.8
*TEM *+* AAC*	3	1.1
*TEM *+* OXA-*1	3	1.1
AmpC-blaCITM	2	0.7
*CTX-M *+* OXA-1 *+ *AAC*	2	0.7
*OXA-*1	2	0.7
*TEM *+* CTX-M *+* FosA*	2	0.7
*TEM *+ *SHV*	2	0.7
*CTX-M *+* AmpC-blaCITM *+ *AAC*	1	0.4
*CTX-M *+* FosA *+* OXA-*1	1	0.4
*CTX-M + SHV *+* qnr*	1	0.4
FosA	1	0.4
KPC	1	0.4
*NDM* +* OXA-*1	1	0.4
*OXA-1 *+* AAC*	1	0.4
*SHV *+* AAC*	1	0.4
*TEM *+ *AmpC-blaCITM*	1	0.4
*TEM *+* AmpC-blaCITM *+* IMP*	1	0.4
*TEM *+ *CTX-M *+* AAC*	1	0.4
*TEM *+ *CTX-M *+* KPC *+ *OXA-1*	1	0.4
*TEM *+* CTX-M *+* OXA-1 *+* AAC*	1	0.4
*TEM *+* CTX-M *+* SHV *+* AAC*	1	0.4
*TEM *+ *CTX-M *+ *SHV *+* AmpC-blaCITM*	1	0.4
*TEM *+* FosA*	1	0.4
*TEM *+* FosA *+ *AmpC-blaCITM*	1	0.4
*TEM *+* IMP*	1	0.4
*TEM *+* KPC*	1	0.4
*TEM *+* KPC + VIM*	1	0.4

Overall, *blaCTX-M* featured in the majority of multidrug resistance profiles, either alone or in combination with other resistance determinants, underscoring its dominant role in ESBL-mediated resistance within this patient population. The frequent co-occurrence of* blaTEM* and *blaCTX-M* suggests co-localization of these genes on mobile genetic elements such as plasmids, facilitating horizontal gene transfer and the dissemination of multidrug-resistant *Enterobacterales* in healthcare settings. The detection of isolates carrying combinations of ESBL genes with *AmpC* β-lactamase genes (e.g., *blaCITM*) and carbapenemase genes (e.g., *KPC, IMP, NDM*, and *VIM*) is of particular clinical concern, despite their low frequency. Such combinations may broaden the spectrum of β-lactam resistance, compromise the effectiveness of last-line antimicrobial agents, and limit therapeutic options for immunocompromised cancer patients.

## Discussion

The most common resistance genes among bacterial isolates recovered from cancer patients at MRRH were *CTX-M *and *TEM. CTX-M* was the most frequent gene, detected in 106/281 (37.7%) isolates, followed by *TEM* in 81/281 (28.8%). At least one target gene was detected in 170/281 (60.5%) isolates, whereas 111/281 (39.5%) had no target gene detected within the PCR panel. This indicates that *CTX-M* and *TEM* were the leading detectable ESBL-associated genes, but also that phenotypic ESBL production in a substantial proportion of isolates may have been mediated by untested ESBL variants or other mechanisms not captured by the PCR targets used.

The predominance of *CTX-M* observed in the present study is consistent with other studies from cancer and non-cancer hospital populations. In Uganda, Lubwamaet al.* *(2024) reported *CTX-M* as the most common ESBL-encoding gene (91%), followed by *TEM* (69%) among *Enterobacterales* isolated from hematological cancer patients with febrile neutropenia and bacteremia at the Uganda Cancer Institute [11]. Similarly, Tohamy et al. reported *CTX-M* as the most common ESBL gene among multidrug-resistant Gram-negative bloodstream isolates from febrile neutropenic cancer patients in Egypt, although *SHV* was more frequent than *TEM* in that study [18]. The current findings are also consistent with findings of a rural southwestern Ugandan study, where* CTX-M* was the dominant ESBL gene among Gram-negative clinical isolates, followed by *TEM* and *SHV* [19].

*E. coli* (155/281; 55.2%) followed by *K. pneumoniae* (85/281; 30.2%) were the most common bacterial isolates in this study. This pattern is biologically plausible because these organisms are common gut colonizers and can become invasive in immunocompromised hosts, especially when mucosal barriers are damaged by chemotherapy, malignancy, prolonged hospitalization, or repeated antibiotic exposure [8]. Similar to this study’s finding, Lubwama et al. 2024 identified *E. coli *to be the predominant *Enterobacterales* isolate among hematological cancer patients with bacteremia in Uganda, while Tohamy et al. reported *E. coli *and *K. pneumoniae *as the leading multidrug-resistant Gram-negative organisms among febrile neutropenic cancer patients with bloodstream infections in Egypt [11,18].

*TEM* + *CTX-M* was the most common co-carriage pattern (25/281; 8.9%). Similar to this co-carriage, Moses et al. in Uganda [19] identified *CTX-M* and *TEM* co-carriage as the second most common multi-gene combination among ESBL-gene-positive isolates at the same institution (28/87; 32.2%), after *CTX-M* and *SHV* co-carriage (36/87; 41.4%). The overall proportions of *CTX*-*M* (37.7%), *TEM* (28.8%), and *SHV* (9.3%) in the present study were lower than those reported by Lubwama et al., who reported *CTX*-*M* in 91%, *TEM* in 69%, and *SHV* in 42% among isolates with ESBL-encoding genes in hematological cancer patients with bacteremia in Uganda [11]. Other higher proportions of these genes have also been reported by Tawfick et al. among pediatric cancer patients in Egypt [20]. These differences are likely due to differences in denominator and case selection because those studies focused on bloodstream infections, hematological malignancies, or highly selected resistant isolates, whereas the present analysis included a broader set of phenotypically confirmed ESBL-producing isolates from multiple specimen types.

The observed differences may also be due to the cancer population used, where studies restricted to hematological malignancy, febrile neutropenia, or bloodstream infection are likely to detect higher resistance levels because these patients often experience profound immunosuppression, mucosal injury, prolonged hospital exposure, and repeated broad-spectrum antibiotic use. As Alevizakos et al. 2016 showed, cancer patients colonized with ESBL-PE were much more likely to develop subsequent bloodstream infection [8]. The present study enrolled a broader mix of cancer types and less severely immunosuppressed patients than hematological febrile-neutropenia cohorts, explaining the lower overall prevalence of individual resistance genes compared with studies limited to high-risk bloodstream infection populations.

The lower frequency of *SHV* observed in the present study may partly reflect differences in bacterial species composition. *SHV* enzymes are more commonly associated with *K. pneumoniae* than with *E. coli*. In the present study, *E. coli *accounted for 155/281 (55.2%) of all isolates, whereas *K. pneumoniae *constituted 85/281 (30.2%). This may have reduced the overall* SHV* proportion. In contrast, Tawfick et al. [20] studied *E. coli* and *K. pneumoniae *isolates and found that *K. pneumoniae*, despite being less numerous than E. coli in their collection, accounted for the largest share of carbapenemase-encoding genes (34/121; 28.1%), a species-driven pattern consistent with the disproportionate *SHV* contribution from* K. pneumoniae* observed in the present study.

There was a relatively low prevalence of carbapenemase-associated genes in this study, suggesting that carbapenemase gene carriage was not yet a dominant molecular feature in this isolate collection. *KPC* was detected in 4/281 (1.4%) isolates, *IMP* in 2/281 (0.7%), and *NDM* and *VIM* in one isolate each (0.4%). This finding is partly similar to earlier work from Uganda, where Ampaire et al. [21] reported carbapenem-resistance genes among multidrug-resistant *Enterobacterales* from MRRH, but only *blaVIM* and *blaOXA*-48 were detected; other important carbapenemase genes, including *blaKPC* and *blaIMP*, were not identified. In this study, co-carriage of multiple resistance genes within individual isolates was also observed, with *CTX-M, TEM,* and other β-lactamase genes occurring together in a subset of isolates. These genes are commonly located on transferable plasmids, transposons, integrons, and other mobile genetic elements that facilitate horizontal gene transfer between bacterial species, thereby promoting the dissemination of antimicrobial resistance within healthcare settings.

However, their overall gene prevalence was higher than in the present study because they first selected multidrug-resistant isolates before testing for carbapenem resistance genes. Therefore, the difference in the denominator is that this study has a lower prevalence of these genes, as this study reports prevalence across all recovered isolates, whereas Ampaire et al. [21] focused on MDR isolates, which are more likely to harbor carbapenemase genes

This present study finding also differs from several studies in cancer and tertiary-care settings where carbapenemase genes were much more frequent. In Egypt, Kamel et al.studied Gram-negative isolates from febrile neutropenic pediatric cancer patients and found high levels of carbapenem resistance and carbapenemase genes, with OXA-48 and NDM being the most frequent. Similarly [22], Tawfick et al. reported that nearly 90% of *Enterobacterales* isolates from Egyptian cancer patients carried one or more carbapenemase genes, with NDM-1 predominating [20]. In Sri Lanka, Chathuranga et al. found that nearly all carbapenem-resistant *Enterobacterales* isolated from adult cancer inpatients carried carbapenemase genes, again with *NDM* as the dominant genotype [23]. The observed differences are due to the current study not being restricted to carbapenem-resistant isolates compared to the other studies that enrich their samples with organisms already under strong antibiotic selection pressure. Lastly, the species composition differs, where the present study was dominated by *E. coli* whereas studies showing high carbapenemase prevalence often focus heavily on *K. pneumoniae*, a species strongly associated with hospital transmission of carbapenemase plasmids and outbreak clones [24,25].

It is important to interpret the above comparisons within the constraints of the cross-sectional design used in this study. Because isolates and their associated resistance genes were characterized at a single point in time for each patient, the study can describe the distribution and relative prevalence of ESBL and other resistance genes among cancer patients at MRRH, but it cannot establish how gene prevalence has changed over time, track the emergence or spread of specific resistance genes or clones within the hospital, or determine whether the resistance-gene profiles observed preceded or resulted from a patient's cancer treatment, antibiotic exposure, or hospitalization. Similarly, associations noted between species, gene families, and patient or specimen characteristics should be read as descriptive patterns rather than causal relationships, since a cross-sectional design cannot separate cause from effect or rule out confounding by unmeasured factors. Longitudinal or repeated-sampling studies would be needed to determine the direction and stability of these associations over time.

Strengths

This study is among the first studies among cancer patients in South-Western Uganda to combine phenotypic ESBL confirmation with targeted molecular characterization of multiple resistance gene families, including blaCTX-M, blaTEM, blaSHV, and carbapenemase-associated genes, in a population with heightened vulnerability to multidrug-resistant infection. An a priori calculated sample size of 281 phenotypically confirmed ESBL-PE isolates was achieved, and isolates were recovered from a broad range of clinical specimen types, including blood, urine, stool, sputum, cerebrospinal fluid, swabs, and aspirates, which improved the representativeness of the isolate collection. Standard operating procedures, internal quality control, and CLSI-guided phenotypic confirmation using the double-disc synergy test were applied throughout isolate recovery, identification, and molecular analysis, strengthening confidence in the reported gene prevalence estimates.

Limitations

This study was conducted at a single referral hospital, Mbarara Regional Referral Hospital, which may limit the generalizability of the findings to other cancer patients and healthcare settings in Uganda. Conventional PCR was used to detect selected resistance genes; therefore, resistance genes, variants, or resistance mechanisms not included in the PCR panel may have been missed, which may partly explain why some phenotypically confirmed ESBL-PE had no target gene detected. Whole-genome sequencing and amplicon sequencing were not performed, limiting the ability to determine the specific allelic variants of the detected resistance genes, identify additional resistance determinants, and assess genetic relatedness among isolates. In addition, plasmid typing was not performed; therefore, the plasmid replicon types, genetic context, and potential mobility of the detected resistance genes could not be established.

Recommendations

MRRH should strengthen routine surveillance for ESBL-PE among cancer patients, with particular attention to *CTX-M* and *TEM*-producing isolates and to phenotypically confirmed ESBL-PE with no target gene detected in the current PCR panel. MRRH clinicians should combine molecular testing with phenotypic antimicrobial susceptibility testing to guide appropriate treatment. Routine monitoring for carbapenemase genes, especially *NDM, KPC, VIM, IMP,* and *OXA*-48-like genes, should be done by MRRH clinical teams to detect early emergence. Future researchers should include multi-centre sampling, clinical risk-factor analysis, and whole-genome sequencing to better understand transmission and resistance evolution in cancer-care settings.

## Conclusions

This study found that ESBL-PE among cancer patients at MRRH were predominantly *E. coli* and *K. pneumoniae*. These findings describe the distribution of ESBL-PE in this population and highlight the importance of continued surveillance of resistant Enterobacterales among patients receiving cancer care. *CTX-M* and *TEM* were the most frequently detected resistance genes. However, 111/281 (39.5%) phenotypically confirmed ESBL-PE isolates had none of the target genes included in the PCR panel, suggesting that other ESBL variants or resistance mechanisms not investigated in this study may contribute to the observed phenotype. Further molecular studies, including broader gene panels and whole-genome sequencing, would help characterize these isolates more comprehensively.

Carbapenemase-associated genes were uncommon among the isolates examined, indicating a low observed prevalence of the carbapenemase genes included in the molecular panel during the study period. Nevertheless, continued surveillance is warranted because the distribution of antimicrobial resistance determinants can change over time. Overall, these findings provide local laboratory-based evidence of the burden and molecular characteristics of ESBL-PE among cancer patients at MRRH. The results may inform antimicrobial stewardship, infection-prevention activities, and future surveillance strategies at the hospital. However, the cross-sectional design does not establish causality, source of infection, or clinical treatment effectiveness. Further prospective and molecular studies are needed to determine the clinical significance, transmission pathways, and broader genetic mechanisms underlying ESBL production in this population.

## Appendices

**Table 6:** Distribution of additional resistance genes (FosA, qnr, blaOXA-1, and AAC) among Enterobacterales The table presents, for quick reference, the additional resistance genes detected at lower frequency in this study and falling outside the primary ESBL and carbapenemase focus of the main text (Table 4)

Bacterial isolate	Total isolates, n	FosA	qnr	OXA-1	AAC
Escherichia coli	155	4 (2.6)	0 (0.0)	6 (3.9)	8 (5.2)
Klebsiella pneumoniae	85	1 (1.2)	0 (0.0)	5 (5.9)	15 (17.6)
Klebsiella oxytoca	15	0 (0.0)	0 (0.0)	1 (6.7)	0 (0.0)
Enterobacter cloacae	15	1 (6.7)	0 (0.0)	0 (0.0)	1 (6.7)
Proteus mirabilis	5	0 (0.0)	1 (20.0)	0 (0.0)	0 (0.0)
Klebsiella aerogenes	3	0 (0.0)	0 (0.0)	0 (0.0)	0 (0.0)
Proteus vulgaris	2	0 (0.0)	0 (0.0)	0 (0.0)	0 (0.0)
Citrobacter freundii	1	0 (0.0)	0 (0.0)	0 (0.0)	0 (0.0)
Overall	281	6 (2.1)	1 (0.4)	12 (4.3)	24 (8.5)
